# Modulation of Host Physiology and Pathophysiology by the Gut Microbiome

**DOI:** 10.3390/nu16030361

**Published:** 2024-01-26

**Authors:** Weston R. Gray, Jonathan P. Jacobs

**Affiliations:** 1 The Vatche and Tamar Manoukian Division of Digestive Diseases, Department of Medicine, David Geffen School of Medicine, University of California Los Angeles, Los Angeles, CA 90095, USA; 2 Goodman-Luskin Microbiome Center, University of California Los Angeles, Los Angeles, CA 90095, USA; 3 Division of Gastroenterology, Hepatology and Parenteral Nutrition, VA Greater Los Angeles Healthcare System, Los Angeles, CA 90073, USA

The human gut microbiome is a highly dynamic community of bacteria, fungi, viruses, archaea, and protozoans that resides within the gastrointestinal tract. With the advent of next-generation sequencing technologies, there is a growing body of evidence highlighting the crucial role of the gut microbiota in host physiology. A balanced and diverse microbiome, a state referred to as eubiosis, not only supports digestion and nutrient absorption, but also plays an integral role in immunoregulation, drug metabolism, and behavior [1,2,3].

Industrialization during the modern era has brought about profound changes to various aspects of human life. The Western diet has seen an influx of processed foods containing high levels of saturated fats and refined sugars and a corresponding decrease in the consumption of dietary fiber [4]. In addition, easier access to broad-spectrum antibiotics has disrupted the balance of the gut microbiota and its functioning. These shifts have led to widespread dysbiosis, a higher prevalence of a diverse array of disease states, and an urgent need to elucidate the mechanistic underpinnings of host–microbiota interactions. This Special Issue of Nutrients, entitled “Modulation of Host Physiology and Pathophysiology by the Gut Microbiome”, features research highlighting the different ways that gut microbiota, acting on dietary and host-derived inputs, affect a variety of health outcomes.

There is a clear connection between diet and pathophysiology that may be mediated by gut microbiota [5]. Several articles in this Special Issue explore the role of microbial short-chain fatty acid production from dietary fiber and how such metabolites influence disease pathogenesis. Fabiano, Shinn, and Antunes conducted an integrative review that evaluated published studies surrounding gut microbiota metabolism of oat fibers, with an emphasis on *β*-glucan (contribution 1). These studies corroborate the prebiotic properties of oat *β*-glucan, demonstrating how its fermentation to short-chain fatty acids could support immunoregulation and the beneficial restructuring of the gut microbiota, among other things. The relationship between gut-microbial-derived short-chain fatty acids and host health is further discussed in a review article by May and Hartigh, which explores SCFA involvement in adipocyte metabolism in the context of obesity (contribution 2). The authors provide a comprehensive, mechanistic view on the effects of short-chain fatty acid signaling in lipid/glucose metabolism, inflammatory responses, and other physiologic processes associated with obesity, while also alluding to the presence of conflicting findings and the need for more longitudinal studies. Ruan and colleagues (contribution 3) also found that a gut-derived SCFA, butyrate, significantly reduces ulcerative colitis (UC) symptoms through the transcriptional modulation of genes involved in anti-inflammatory responses. The treatment of UC through the exogenous administration of a single species of butyrate-producing bacteria, *R. intestinalis*, in a murine colitis model sheds light on the therapeutic potential of short-chain fatty acids and the commensals that produce them.

Interventions such as prebiotics and probiotics can be used to shape the gut microbiota for potential therapeutic benefit. The review article by Chernikova, Zhao, and Jacobs (contribution 4) delves into the role of the microbiome in food allergies and potential avenues for modulating the microbiome to prevent and treat this condition, including dietary modification as well as prebiotics and probiotics. The authors discuss the role of the bacterial synthesis of SCFAs, secondary bile acids, sphingolipids, and tryptophan metabolites such as indole-3-aldehyde in food allergy development, while also touching on relevant targeted microbial therapies and clinical trials evaluating their efficacy. The therapeutic potential of probiotic supplementation is further explored by Ibrahim and colleagues, who investigated the impact of *B. amyloliquefaciens*-supplemented camel milk yogurt (BASY) in a murine model of multiple sclerosis (contribution 5). BASY administration increased microbial load in diseased mice, subsequently reducing pathological signs of multiple sclerosis, including demyelination and paralysis, in addition to molecular proxies of systemic inflammation.

Taken together, the studies presented in this Special Issue of Nutrients are a testament to the importance of the human gut microbiome in the way diseases are conceptualized and treated. The symbiosis between humans and the gut microbiota can be harnessed to prevent, detect, and ameliorate biological dysfunction. Though current research efforts are met with a number of challenges, the aforementioned studies will undoubtedly pave the path for a greater understanding of the role of the microbiome in human health and diseases.
